# Genetic Polymorphism of Apolipoprotein A5 Gene and Susceptibility to Type 2 Diabetes Mellitus: A Meta-Analysis of 15,137 Subjects

**DOI:** 10.1371/journal.pone.0089167

**Published:** 2014-02-19

**Authors:** Yan-Wei Yin, Qian-Qian Sun, Pei-Jian Wang, Li Qiao, Ai-Min Hu, Hong-Li Liu, Qi Wang, Zhi-Zhen Hou

**Affiliations:** 1 Department of Emergency, Chinese PLA Air Force General Hospital, Haidian District, Beijing, China; 2 Jinsong Sanatorium of Beijing Air Force, Beijing, China; 3 Department of Cardiology, the First Affiliated Hospital, Chengdu Medical College, Chengdu, Sichuan, China; 4 Department of Dermatology, Chinese PLA Air Force General Hospital, Haidian District, Beijing, China; National Cancer Center, Japan

## Abstract

**Background:**

Several studies have investigated whether the polymorphism in the apolipoprotein A5 (APOA5) is associated with type 2 diabetes mellitus (T2DM) risk. However, those studies have produced inconsistent results. The purpose of this study was to investigate whether the APOA5 -1131T/C polymorphism (rs662799) confers significant susceptibility to T2DM using a meta-analysis.

**Methods:**

PubMed, Embase, Web of Science, Cochrane database, CBMdisc, CNKI and Google Scholar were searched to get the genetic association studies. All statistical analyses were done with Stata 11.0.

**Results:**

A total of 19 studies included 4,767 T2DM cases and 10,370 controls (four studies involving 555 T2DM cases and 2958 controls were performed among Europeans and 15 studies involving 4212 T2DM cases and 7412 controls were performed among Asians) were combined showing significant association between the APOA5 -1131T/C polymorphism and T2DM risk (for C allele vs. T allele: OR = 1.28, 95% CI = 1.17–1.40, *p*<0.00001; for C/C vs. T/T: OR = 1.57, 95% CI = 1.35–1.83, *p*<0.00001; for C/C vs. T/C+T/T: OR = 1.36, 95% CI = 1.18–1.57, *p*<0.0001; for C/C+T/C vs. T/T: OR = 1.32, 95% CI = 1.16–1.51, *p*<0.0001). In the subgroup analysis by ethnicity, significant association was also found among Asians (for C allele vs. T allele: OR = 1.31, 95% CI = 1.22–1.40, *p*<0.00001; for C/C vs. T/T: OR = 1.61, 95% CI = 1.38–1.88, *p*<0.00001; for C/C vs. T/C+T/T: OR = 1.39, 95% CI = 1.20–1.61, *p*<0.0001; for C/C+T/C vs. T/T: OR = 1.42, 95% CI = 1.25–1.62, *p*<0.00001). However, no significant association was found between the APOA5 -1131T/C polymorphism and T2DM risk among Europeans.

**Conclusions:**

The present meta-analysis suggests that the APOA5 -1131T/C polymorphism is associated with an increased T2DM risk in Asian population.

## Introduction

Type 2 diabetes mellitus (T2DM) is a common, multifactorial disease that causes significant morbidity and mortality worldwide [1], [2]. Although it results from synthetic action involving insulin resistance and impaired insulin secretion [3], a detailed etiology underlying T2DM is still unclear. Meanwhile, T2DM is a major factor that along with other risk factors such as obesity, endothelial dysfunction, and dyslipidemia contribute to atherosclerotic diseases. Therefore, it is vitally important to investigate the pathogenesis of T2DM.

Recently, numerous epidemiological studies have focused on the associations between the apolipoprotein A5 (APOA5) gene polymorphisms and T2DM risk, and indicate that the APOA5 gene polymorphisms exert important role in the development of T2DM [4]–[22]. APOA5 is a newly discovered member of the APOA4/APOC3/APOA1 apolipoprotein cluster [23], [24]. The human APOA5 gene is located at chromosome 11q23 [20], displays a few polymorphisms in the promoter region (e.g., -1131T>C, -3A>G, S19W, IVS3+476G>A, 1259T>C, and G185C), which associated with high plasma triglyceride (TG) levels [23], [25]–[28] and TG-related diseases such as metabolic syndrome [29], atherosclerotic diseases [30] and Alzheimer's disease [31]. Previous meta-analyses have reported that the APOA5 -1131T/C polymorphism is associated with an increased risk for developing metabolic syndrome [32], [33]. Here, we focused on the association between the APOA5 -1131T/C polymorphism and T2DM due to rare studies on other single-nucleotide polymorphisms. Until now, whether the APOA5 -1131T/C polymorphism is related to the risk of T2DM is still under debate. Most studies reported that the APOA5 -1131T/C polymorphism was associated with an increased risk of T2DM [6], [7], [11], [12], [14]–[16], [19]. However, other studies demonstrated that there was no significant association between the APOA5 -1131T/C polymorphism and T2DM risk [4], [5], [8], [9], [17], [18], [20]. Therefore, to better clarify the association between the APOA5 -1131T/C polymorphism and T2DM risk, we conducted a meta-analysis by collecting and sorting the previous published studies.

## Materials and Methods

### Literature search

We performed this meta-analysis in accordance with the Preferred Reporting Items for Systematic Reviews and Meta-analyses (PRISMA) criteria [34]. Eligible literatures published before the end of October 2013 were identified by the search of PubMed, Embase, Web of Science, Cochrane database, CBMdisc, CNKI and Google Scholar using combinations of the following keywords: (apolipoprotein A5 OR APOA5 OR rs662799) AND (“polymorphism” OR “mutation” OR “variant” OR “variation” OR “genotype”) AND (type 2 diabetes mellitus OR type 2 diabetes OR diabetes mellitus OR diabetic patients OR T2DM). Furthermore, genome-wide association studies (GWAS) for T2DM were also identified by searching the above databases. Finally, the references of all selected studies were examined to identify additional work not indexed by electronic databases. There was no restriction on time period, sample size, population, or language.

### Inclusion criteria

The studies included in the meta-analysis must meet all the following inclusion criteria: (1) evaluation of the association between APOA5 -1131T/C polymorphism and T2DM risk; (2) the design had to be a case-control study or cohort study; (3) sufficient published data for calculating odds ratios (ORs) with their 95% confidence intervals (CIs); and (4) not republished data.

### Data extraction

For each study, the following information was extracted: name of the first author, year of publication, study population (country, ethnicity), source of controls (population-based studies and hospital-based studies), sample size (total numbers of cases and controls), and genotype frequency in cases and controls. Two authors (Yin YW and Sun QQ) independently assessed the articles for compliance with the inclusion criteria, resolved disagreements and reached a consistent decision.

### Quality score assessment

To determine the methodological quality of each study, we used the Newcastle-Ottawa scale (NOS), which uses a “star” rating system to judge the quality of observational studies [35]. Three aspects of the study were assessed (selection, comparability, and exposure), and each satisfactory answer received one star. The NOS ranges between zero (worst) up to nine stars (best). Studies with a score of seven stars or greater are considered to be of high quality. Two authors (Liu HL and Wang Q) independently assessed the quality of included studies, and the result was reviewed by a third author (Wang Q). The discrepancy in quality assessment was discussed and resolved by the three authors.

### Statistical analysis

The strength of the association between the APOA5 -1131T/C polymorphism and T2DM risk was evaluated by the ORs with 95% CIs under the allelic model (C allele vs. T allele), additive model (C/C vs. T/T), recessive model (C/C vs. T/C+T/T), and dominant model (C/C+T/C vs. T/T). To adjust for multiple comparisons, we applied the Bonferroni method, which control for false positive error rate (*p*<0.0125 was considered to be statistically significant). Hardy-Weinberg equilibrium (HWE) in the control group was assessed using the chi-square test (*p*<0.05 was considered significant deviation from HWE).

Cochran's Q statistic and the I^2^ statistic were performed to evaluate possible heterogeneity (*p*<0.10 and I^2^>50% indicated evidence of heterogeneity) [36], [37]. A random-effects model or fixed-effects mode was used to calculate pooled OR in the presence or absence of heterogeneity, respectively. The sources of heterogeneity were also investigated by subgroup and sensitivity analyses. Subgroup analysis was performed based on ethnicity (Europeans and Asians). Sensitivity analyses were performed based on HWE (studies with HWE were included), NOS score (studies with score ≥7 were included), and source of controls (studies with population-based controls were included). Furthermore, Galbraith plot was used to further detect the potential sources of heterogeneity. Finally, publication bias was assessed by Begg's funnel plot [38] and Egger's regression test [39] (*p*<0.05 was considered representative of statistically significant publication bias). Data analyses were performed using Stata software (Version 11; StataCorp LP, College Station, TX) and Review Manager software 5.1.4 (Cochrane Collaboration, The Nordic Cochrane Centre, Copenhagen).

## Results

### Characteristics of the studies

**Figure 1:**
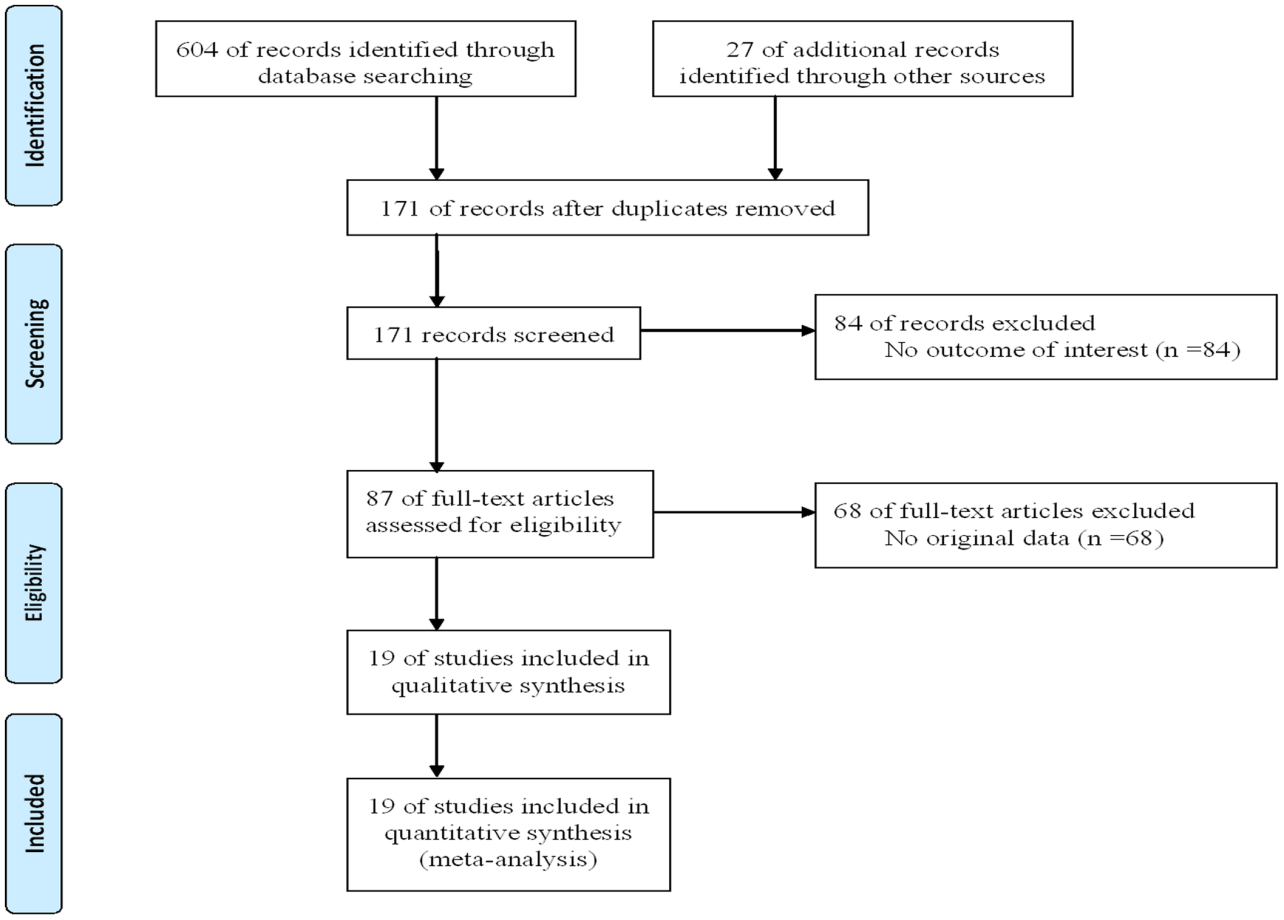
Flow diagram of the study selection process.

The present study met the PRISMA statement requirements (Checklist S1 and Fig. 1). The initial search identified 631 potentially eligible articles. Due to the high repetition rate of article in different databases, 460 articles were then excluded by reading the title. After reading the abstract and full text, 152 irrelevant articles were excluded. Finally, 19 eligible articles involving 4,767 T2DM cases and 10,370 controls were identified to assess the association of the APOA5 -1131T/C polymorphism with T2DM risk [4]–[22]. The baseline characteristics of 19 studies were shown in Table 1 and 2. Most studies were carried out among Asians and four studies were carried out among Europeans. Five studies did not follow the HWE [9], [12], [15], [18], [20] and one study had insufficient data for calculation of the HWE [22]. The NOS results showed that the average score was 7.6, which indicated that the methodological quality was generally good.

**Table 1:** Characteristics of studies included in this meta-analysis.

				Source of Controls	Sample size (case/control)	Genotypes distribution (case/control)					HWE	
First author	Year	Country	Ethnicity			T/T	T/C	C/C	T	C	Y/N (P)	Score
Li [4]	2002	China	Asian	PB	214/472	96/225	85/196	33/51	277/646	151/298	Y(0.398)	8
Chaaba [5]	2005	Tunisia	European	HB	152/156	108/115	38/36	6/5	254/266	50/46	Y(0.305)	6
Yan [6]	2005	China	Asian	PB	285/155	117/83	139/58	29/14	373/224	197/86	Y(0.407)	8
Liu [7]	2005	China	Asian	PB	465/502	171/246	225/212	69/44	567/704	363/300	Y(0.861)	8
Talmud [8]	2006	UK	European	PB	142/2348	131/2067	11/267	0/14	273/4401	11/295	Y(0.097)	6
Baum [9]	2007	China	Asian	PB	749/198	373/109	283/65	93/24	1029/283	469/113	N(0.006)	8
Cheng [10]	2007	China	Asian	PB	275/113	108/57	139/48	28/8	355/162	195/64	Y(0.623)	9
Zhai [11]	2007	China	Asian	PB	71/152	21/77	39/60	11/15	81/214	61/90	Y(0.514)	8
Zhou [12]	2008	China	Asian	PB	222/150	74/79	107/49	41/22	255/207	189/93	N(0.004)	8
Feng [13]	2008	China	Asian	PB	130/175	50/86	69/79	11/10	169/251	91/99	Y(0.136)	8
Qiao [14]	2008	China	Asian	PB	154/206	85/117	52/79	17/10	222/313	86/99	Y(0.470)	8
Li [15]	2008	China	Asian	PB	476/340	188/172	198/124	90/44	574/468	378/212	N(0.006)	9
Zhang [16]	2009	China	Asian	PB	484/538	212/294	230/210	42/34	654/798	314/278	Y(0.667)	8
Li [17]	2009	China	Asian	PB	45/40	30/27	9/10	6/3	69/64	21/16	Y(0.166)	7
Xiao [18]	2010	China	Asian	PB	54/108	26/54	16/35	12/19	68/143	40/73	N(0.004)	8
Bhaskar [19]	2011	India	Asian	PB	310/120	151/70	130/41	29/9	432/181	188/59	Y(0.389)	7
Sóter [20]	2012	Brazil	European	PB	146/173	111/134	34/33	1/6	256/301	36/45	N(0.039)	7
Yao [21]	2012	China	Asian	HB	278/4143	139/2065	116/1705	23/373	394/5835	162/2451	Y(0.434)	7
						T/T	T/C+C/C					
Can Demirdöğen [22]	2012	Turkey	European	HB	115/281	97/221	18/60				—	7

PB: population-based, HB: hospital-based.

HWE: Hardy-Weinberg equilibrium, Y: yes, N: no.

**Table 2:** Meta-analyses of APOA5 -1131T/C polymorphism and risk of T2DM in each subgroup.

	Sample size (case/control)	Allelic model			Additive model			Recessive model			Dominant model		
Position		OR(95% CI)	P	P_Q_, I^2^(%)	OR(95% CI)	P	P_Q_, I^2^(%)	OR(95% CI)	P	P_Q_, I^2^(%)	OR(95%CI)	P	P_Q_, I^2^(%)
Overall analysis													
-1131T/C	4767/10370	1.28[1.17,1.40]^a^	<0.00001	0.05(38%)	1.57[1.35,1.83]	<0.00001	0.37(7%)	1.36[1.18,1.57]	<0.0001	0.69(0%)	1.32[1.16,1.51]^a^	<0.0001	0.004(52%)
Subgroup analysis based on ethnicity													
-1131T/C(E)	555/2958	0.91[0.69,1.20]	0.49	0.25(28%)	0.68[0.27,1.70]	0.41	0.31(14%)	0.66[0.27,1.66]	0.38	0.31(15%)	0.88[0.67,1.15]	0.34	0.31(17%)
-1131T/C(A)	4212/7412	1.31[1.22,1.40]	<0.00001	0.15(28%)	1.61[1.38,1.88]	<0.00001	0.46(0%)	1.39[1.20,1.61]	<0.0001	0.77(0%)	1.42[1.25,1.62]^a^	<0.00001	0.04(43%)
Sensitivity analysis													
-1131T/C(BH)	3005/9120	1.27[1.14,1.42]^a^	<0.0001	0.05(42%)	1.61[1.34,1.93]	<0.00001	0.49(0%)	1.41[1.18,1.68]	<0.0001	0.76(0%)	1.33[1.14,1.56]^a^	0.0003	0.02(51%)
-1131T/C(BS)	4473/7866	1.30[1.21,1.39]	<0.00001	0.13(30%)	1.58[1.36,1.84]	<0.00001	0.29(14%)	1.37[1.18,1.58]	<0.0001	0.58(0%)	1.37[1.20,1.56]^a^	<0.00001	0.01(49%)
-1131T/C(BC)	4222/5790	1.34[1.24,1.44]	<0.00001	0.26(16%)	1.69[1.44,1.99]	<0.00001	0.70(0%)	1.44[1.23,1.68]	<0.00001	0.83(0%)	1.42[1.25,1.61]^a^	<0.00001	0.06(38%)

A: Asians, E: Europeans.

BH: based on HWE (studies with HWE were included).

BS: based on NOS score (studies with score ≥7 were included).

BC: based on source of controls (studies with population-based controls were included).

a Significant heterogeneity: the random-effects model was chosen to summarize the result.

P_Q_: P values for heterogeneity from Q-test.

### Quantitative synthesis

**Figure 2:**
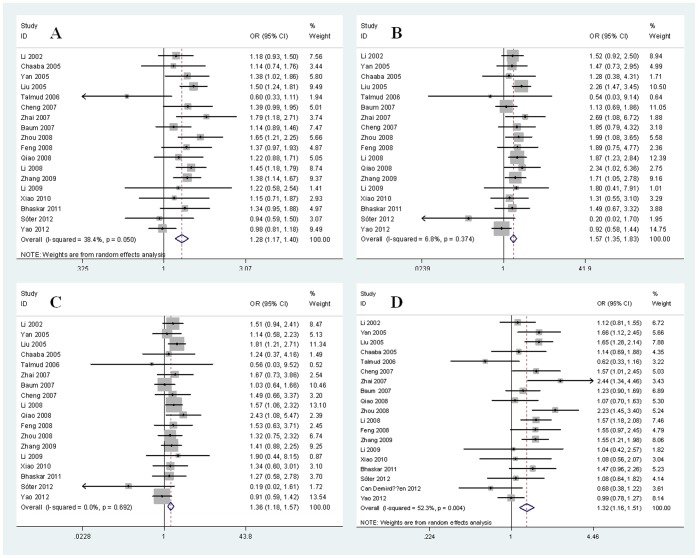
Forest plots for APOA5 -1131T/C polymorphism and T2DM risk. (A). Allelic model: C allele vs. T allele; (B). Additive model: C/C vs. T/T (C). Recessive model: C/C vs. T/C+T/T; (D). Dominant model: C/C+T/C vs. T/T.

Given that significant heterogeneity existed in the allelic model and dominant model, we chose the random-effects model to synthesize the data of these two genetic models. As for the additive model and recessive model, no heterogeneity was found in these two genetic models. Therefore, we used fixed-effects model to analyze the data of additive and recessive models. The overall results showed evidence of significant association between the APOA5 -1131T/C polymorphism and T2DM risk, suggesting that the APOA5 -1131T/C polymorphism was a risk factor for T2DM (for C allele vs. T allele: OR = 1.28, 95% CI = 1.17–1.40, *p*<0.00001; for C/C vs. T/T: OR = 1.57, 95% CI = 1.35–1.83, *p*<0.00001; for C/C vs. T/C+T/T: OR = 1.36, 95% CI = 1.18–1.57, *p*<0.0001; for C/C+T/C vs. T/T: OR = 1.32, 95% CI = 1.16–1.51, *p*<0.0001). The main results of meta-analysis were shown in Table 2 and Fig. 2.

In the subgroup analysis based on ethnicity, significant association was also found between the APOA5 -1131T/C polymorphism and T2DM risk in Asians (for C allele vs. T allele: OR = 1.31, 95% CI = 1.22–1.40, *p*<0.00001; for C/C vs. T/T: OR = 1.61, 95% CI = 1.38–1.88, *p*<0.00001; for C/C vs. T/C+T/T: OR = 1.39, 95% CI = 1.20–1.61, *p*<0.0001; for C/C+T/C vs. T/T: OR = 1.42, 95% CI = 1.25–1.62, *p*<0.00001), but not in Europeans (for C allele vs. T allele: OR = 0.91, 95% CI = 0.69–1.20, *p* = 0.49; for C/C vs. T/T: OR = 0.68, 95% CI = 0.27–1.70, *p* = 0.41; for C/C vs. T/C+T/T: OR = 0.66, 95% CI = 0.27–1.66, *p* = 0.38; for C/C+T/C vs. T/T: OR = 0.88, 95% CI = 0.67–1.15, *p* = 0.34). The main results of subgroup analysis were shown in Table 2.

### Sensitivity analysis

Sensitivity analyses were respectively performed based on HWE (studies with HWE were included), NOS score (studies with score ≥7 were included), and source of controls (studies with population-based controls were included). Overall, the pooled ORs and 95% CIs were not materially altered when any part of the study was omitted, indicating that our results were statistically robust. The results of sensitivity analyses were shown in Table 2.

### Heterogeneity analysis

**Figure 3:**
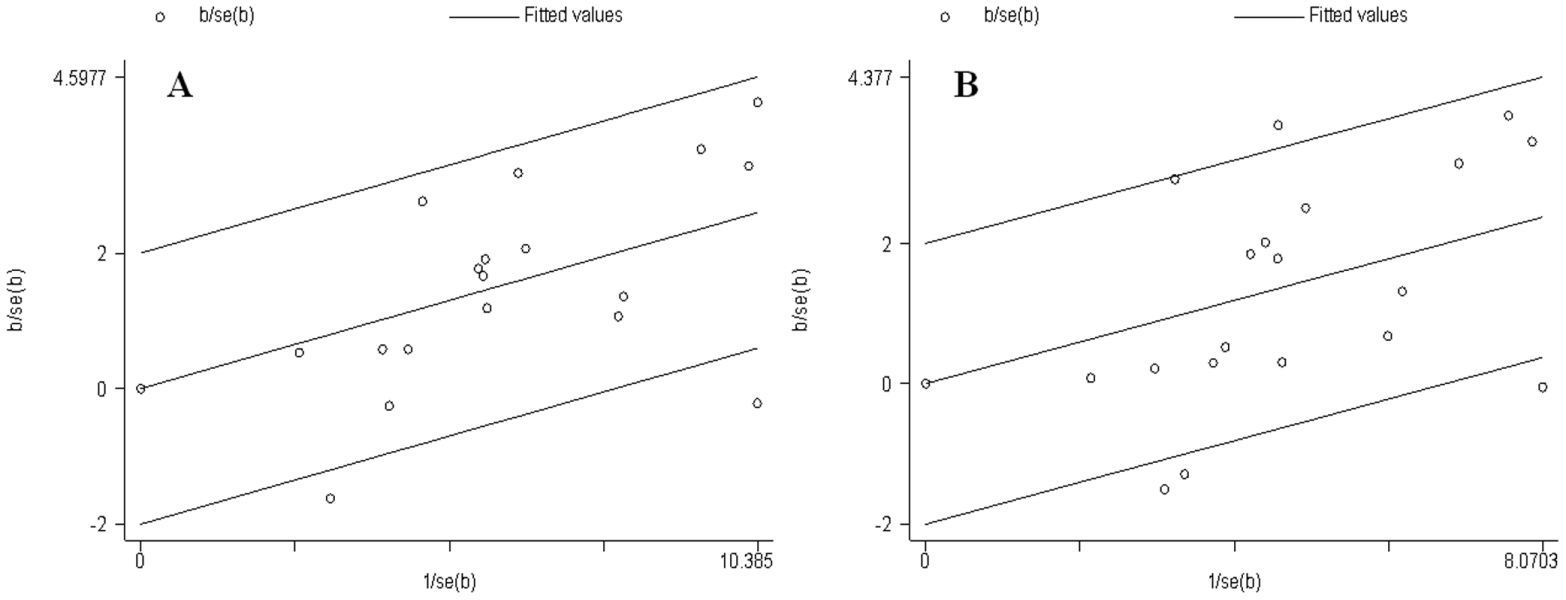
Galbraith plots for APOA5 -1131T/C polymorphism and T2DM risk. (A). Allelic model: C allele vs. T allele; (B). Dominant model: C/C+T/C vs. T/T.

Significant heterogeneity existed in the allelic model (P_Q_ = 0.05, I^2^ = 38%) and dominant model (P_Q_ = 0.004, I^2^ = 52%). In contrast, the additive model (P_Q_ = 0.37, I^2^ = 7%) and recessive model (P_Q_ = 0.69, I^2^ = 0%) did not present significant heterogeneity. To clarify the sources of heterogeneity, we first conducted the subgroup and sensitivity analyses. We only effectively removed the heterogeneity in the European group. We next created a Galbraith plot to graphically assess the sources of heterogeneity. A total of four studies [8], [12], [21], [22] were identified as the main contributors to heterogeneity (Fig. 3). After excluding the outlier studies, the heterogeneity was effectively removed (data not shown).

### Publication bias evaluation

**Figure 4:**
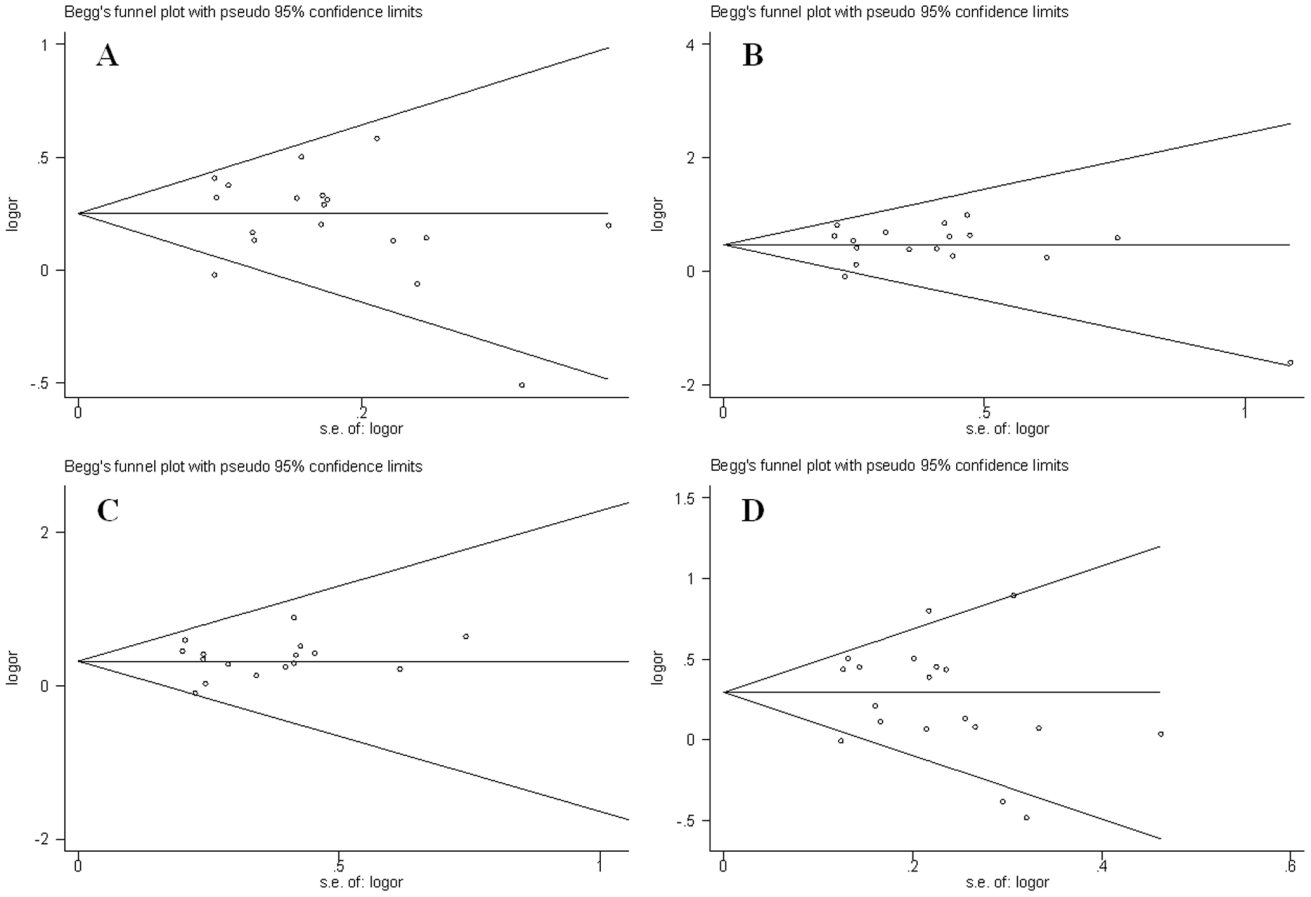
Funnel plots for APOA5 -1131T/C polymorphism and T2DM risk. (A). Allelic model: C allele vs. T allele; (B). Additive model: C/C vs. T/T (C). Recessive model: C/C vs. T/C+T/T; (D). Dominant model: C/C+T/C vs. T/T.

Begg's funnel plot and Egger's regression test were performed to assess potential publication bias. The funnel plot did not show obvious asymmetry in any genetic model (Fig. 4). In addition, the results of Egger's regression test did not provide any statistical evidence for publication bias (*p* = 0.475 for allelic model, *p* = 0.647 for additive model, *p* = 0.581 for recessive model, and *p* = 0.452 for dominant model). Therefore, there was no risk of publication bias in the present meta-analysis.

## Discussion

The APOA5 gene was identified by Pennacchio et al. in 2001 [23]. Since then, the association of APOA5 -1131T/C polymorphism with the susceptibility to T2DM has been widely studied; however, the results are conflicting [4]–[22]. Moreover, the credibility of results from a single case-control study is questionable due to too small sample size of the study populations. Recently, meta-analysis has been widely used in genetic association studies beacuse it has the potential to detect small effects between gene polymorphism and human disease [40], [41]. A previous meta-analysis has reported that the APOA5 -1131T/C polymorphism is associated with an increased T2DM risk [42]. Consistently, the present meta-analysis obtained the similar conclusion. However, only five studies [6], [8], [11], [14], [15] were included in the previous meta-analysis and thus, unable to provide enough persuasiveness. Therefore, a large-scale meta-analysis evaluating the precise association between the APOA5 -1131T/C polymorphism and T2DM risk is required.

To the best of our knowledge, this is the first comprehensive meta-analysis to date investigating the association between the APOA5 -1131T/C polymorphism and T2DM risk. Nineteen studies involving 4,767 T2DM cases and 10,370 controls were included in the present meta-analysis. The overall results showed that there was significant association between the APOA5 -1131T/C polymorphism and the T2DM risk, suggesting the C allele was a independent risk factor for the development of T2DM. The findings showed that the risk of developing T2DM in C allele carriers was 1.32-fold higher than those without. Furthermore, the individuals with C/C genotype had a significantly higher risk for developing T2DM (for OR = 1.57 in additive model and OR = 1.36 in recessive model) compared to those with T/C genotype and T/T genotype.

Considering that potential ethnic difference might be associated with the distribution of genotypes, we also performed subgroup analysis by ethnicity of study population. We only obtained the positive result in the Asians, which further consolidated the overall results. For Europeans, we did not find significant association between the APOA5 -1131T/C polymorphism and T2DM risk. Actually, the minor allele frequency (MAF) of APOA5 -1131T/C is very low among utah residents with Northern and Western European ancestry (CEU) (MAF = 0.015 in HapMap CEU) [43]. However, the APOA5 -1131T/C polymorphism is common among Han Chinese in Beijing, China (CHB) and Japanese in Tokyo, Japan (JPT) (MAF = 0.267 and 0.291, respectively) [43]. Compared with the overall results, the inconsistent results of European group may partly result from genetic diversity among ethnicities. In addition, the number of the studies and the number of European subjects included in this meta-analysis are relatively small (four studies involving 555 T2DM cases and 2958 controls), which could increase the probability of false negatives. Therefore, the results of European group should be interpreted with caution. In addition to the subgroup analysis, sensitivity analyses were also conducted to ascertain whether modification of the inclusion criteria of the meta-analysis affected the final results. The corresponding pooled ORs were not materially altered in all comparisons. The results of sensitivity analyses indicated that our overall results were statistically robust. Furthermore, they also suggested that the studies with hospital-based controls, low NOS score or without HWE should be not considered as a factor influencing the overall results.

In the present meta-analysis, we found significant association between the APOA5 -1131T/C polymorphism and T2DM risk, particularly in Asian population. However, so far no definite evidence exists to support that the APOA5 -1131T/C polymorphism is directly related to T2DM susceptibility. It is well known that high plasma TG level is an important risk factor in developing T2DM [44]. In 2012, Wang et al. found that the baseline TG level was independently associated with T2DM onset risk, and the change of TG level in a 15-year interval predicts the onset risk of T2DM beyond the baseline TG level [44]. Given the large genetic effect of the APOA5 -1131T/C polymorphism on plasma TG level, the observed association between this variant and T2DM might be due to the correlation between the plasma TG level and T2DM risk. Therefore, projections from the literature of who is at risk for APOA5 gene attributable T2DM and who would benefit from APOA5 gene-targeted therapies should be approached with caution.

Between-study heterogeneity should not be ignored and should be carefully factored in the interpretation of the finally results [45], which was also observed in our meta-analysis regarding the allelic model and dominant model. After subgroup and sensitivity analyses, we effectively removed the heterogeneity in the European group. Therefore, the heterogeneity might partly result from the ethnicity difference or the Asian group. However, it should be noted that only four studies involving European subjects were included in the present meta-analysis, which could increase the probability of false positives. Therefore, this result of heterogeneity analysis should be interpreted with caution. In addition to the European group, the heterogeneity in some comparison groups remained significant. To further explore the sources of heterogeneity, we created a Galbraith plot to identify potential outlier studies. A total of four studies [8], [12], [21], [22] were identified as the main contributors to heterogeneity (Fig. 3). After excluding the outlier studies, the heterogeneity was effectively removed in the allelic model and dominant model. Furthermore, the corresponding pooled ORs were not materially altered in all comparisons, suggesting the overall results of this meta-analysis were statistically robust.

For better interpreting the results, some limitations of this meta-analysis should be acknowledged. Firstly, there was significant between-study heterogeneity in the allelic model and dominant model. Heterogeneity is a problem that may affect the precision of overall results. Secondly, in 19 studies included in this meta-analysis, only four of them are European samples, which may develop a partial result. Thirdly, the controls were not uniformly defined. Both healthy individuals and patients without T2DM in hospital were included in the control group. The hospital-based controls may not be representative of the randomized population. Therefore, some inevitable selection bias might be brought into the meta-analyses, which might affect the interpretation of the final results. Moreover, meta-analysis is a type of retrospective study, and recall and selection bias might inevitably exist.

In conclusion, the present meta-analysis suggests that the APOA5 -1131T/C polymorphism is associated with an increased T2DM risk in Asian population. However, the result should be interpreted with caution because of its limitations. Larger and well-designed studies based on different ethnic samples are needed to confirm our results in the future.

## Supporting Information

Checklist S1 **PRISMA 2009 checklist.**
(DOC)
