# Supplementary material for: Genetic Polymorphism of Apolipoprotein A5 Gene and Susceptibility to Type 2 Diabetes Mellitus: A Meta-Analysis of 15,137 Subjects
Source: PLoS One. 2014 Feb 19;9(2):e89167. doi: 10.1371/journal.pone.0089167 (PMC3929635; doi:10.1371/journal.pone.0089167)
Supplement: Checklist S1 — PRISMA 2009 checklist. (DOC) [file pone.0089167.s001.doc]

| **Section/topic** | **#** | **Checklist item** | **Reported on page #** |
| --- | --- | --- | --- |
| **TITLE** | | |  |
| Title | 1 | Genetic polymorphism of apolipoprotein A5 gene and susceptibility to type 2 diabetes mellitus: A meta-analysis of 15,137 subjects | Title |
| **ABSTRACT** | | |  |
| Structured summary | 2 | **Background:** Several studies have investigated whether the polymorphism in the apolipoprotein A5 (APOA5) is associated with the risk of type 2 diabetes mellitus (T2DM). However, those studies have produced mixed results. The purpose of this study is to investigate whether the APOA5 -1131T/C polymorphism confer signiﬁcant susceptibility to T2DM using a meta-analysis.  **Methods:** PubMed, Embase, Web of Science, Cochrane database, CBMdisc, CNKI and Google Scholar were searched to get the genetic association studies. All statistical analyses were done with Stata 11.0.  **Results:** A total of 19 studies included 4,767 T2DM cases and 10,370 controls were combined showing significant association between the APOA5 -1131T/C polymorphism and T2DM risk (for C allele vs. T allele: OR = 1.28, 95% CI = 1.17-1.40, p < 0.01; for C/C vs. T/T: OR = 1.57, 95% CI = 1.35-1.83, p < 0.01; for C/C vs. T/C+T/T: OR = 1.36, 95% CI = 1.18-1.57, p < 0.01; for C/C+T/C vs. T/T: OR = 1.32, 95% CI = 1.16-1.51, p < 0.01). In the subgroup analysis by ethnicity, significant association was also found among Asians (for C allele vs. T allele: OR = 1.31, 95% CI = 1.22-1.40, p < 0.01; for C/C vs. T/T: OR = 1.61, 95% CI = 1.38-1.88, p < 0.01; for C/C vs. T/C+T/T: OR = 1.39, 95% CI = 1.20-1.61, p < 0.01; for C/C+T/C vs. T/T: OR = 1.42, 95% CI = 1.25-1.62, p < 0.01).  **Conclusions:** The present meta-analysis suggests that the APOA5 -1131T/C polymorphism is associated with an increased T2DM risk. | Abstract |
| **INTRODUCTION** | | |  |
| Rationale | 3 | Recently, numerous epidemiological studies have focused on the association between the apolipoprotein A5 (APOA5) -1131T/C polymorphism and T2DM risk, and indicated that the APOA5 -1131T/C polymorphism exerted important role in the development of T2DM [4-22]. Most studies reported that the APOA5 -1131T/C polymorphism was associated with an increased risk of T2DM [6,7,11,12,14-16,19]. However, other studies demonstrated that there was no significant association between the APOA5 -1131T/C polymorphism and T2DM risk [4,5,8,9,17,18,20]. | Introduction |
| Objectives | 4 | Previous meta-analyses have reported that the APOA5 -1131T/C polymorphism is associated with an increased risk for developing coronary artery disease (CAD) and ischemic stroke (IS) [32,33]. However, whether the APOA5 -1131T/C polymorphism is related to the risk of T2DM is still under debate. To better clarify the association between the APOA5 -1131T/C polymorphism and T2DM risk, we conducted a meta-analysis by collecting and sorting the previous published studies. | Introduction |
| **METHODS** | | |  |
| Protocol and registration | 5 | No protocol and registration. |  |
| Eligibility criteria | 6 | To be included in the present meta-analysis, the studies had to comply with the following major criteria: (1) evaluates the association of APOA5 polymorphism with T2DM risk; (2) uses case–control or cohort design; (3) sufficient published data for calculating odds ratios (ORs) with their 95% confidence intervals (CIs); and (4) not republished data. | Inclusion criteria |
| Information sources | 7 | Eligible literatures published before the end of August 2013 were identified by the search of PubMed, Embase, Web of Science, Cochrane database, CBMdisc, CNKI and Google Scholar. In addition, all references cited were reviewed to identify additional studies. | Literature search |
| Search | 8 | Following Medical Subject Heading (MeSH) terms and/or text words were used for searching: (apolipoprotein A5 OR APOA5) AND (“polymorphism” OR “mutation” OR “variant” OR “variation” OR “genotype”) AND (type 2 diabetes mellitus OR type 2 diabetes OR diabetes mellitus OR diabetic patients OR T2DM). In addition, all references mentioned in the identified original articles were reviewed by hand-searching in order to investigate additional literature that was not indexed. | Literature search |
| Study selection | 9 | The flow diagram of the study selection for this meta-analysis was shown in the Fig. 1. By screening title or abstract and further evaluating full-text, we identified 19 eligible articles from PubMed, Embase, Web of Science, Cochrane database, CBMdisc, CNKI and Google Scholar. In total, 4,767 T2DM cases and 10,370 controls were identified to assess the association of the variant with T2DM risk. | Characteristics of the studies |
| Data collection process | 10 | Data were independently extracted from original publications by two reviewers (Yin YW and Sun QQ) according to the inclusion criteria listed above. Discrepancy between the reviewers was resolved by consensus or a third reviewer (Qiao L). | Data extraction |
| Data items | 11 | Data, including name of the first author, year of publication, study population (country, ethnicity), source of controls (population-based studies and hospital-based studies), sample size (total numbers of cases and controls), and genotype frequency in cases and controls, were extracted from each study. | Data extraction |
| Risk of bias in individual studies | 12 | Publication bias was assessed by Begg’s funnel plot and Egger’s regression test (p < 0.05 was considered representative of statistically significant publication bias) | Statistical analysis |
| Summary measures | 13 | The principal summary measures are odds ratios(ORs) and 95% confidence intervals (CIs). | Statistical analysis |
| Synthesis of results | 14 | The combined ORs were respectively calculated for allelic model (C allele vs. T allele), additive model (C/C vs. T/T), recessive model (C/C vs. T/C+T/T), and dominant model (C/C+T/C vs. T/T). Heterogeneity between studies was formally tested by using Cochran’s Q statistic and considered statistically signiﬁcant when p＜0.10. Heterogeneity was also measured with I^2^ statistic (I^2^>50% indicated evidence of heterogeneity). The fixed-effects model was used in the absence of between-study heterogeneity; otherwise the random-effects model was used. | Statistical analysis |

Page 1 of 2

| **Section/topic** | **#** | **Checklist item** | **Reported on page #** |
| --- | --- | --- | --- |
| Risk of bias across studies | 15 | An estimate of potential publication bias was carried out by Begg’s funnel plot and Egger’s regression test (p < 0.05 was considered representative of statistically signiﬁcant publication bias) | Statistical analysis |
| Additional analyses | 16 | Subgroup analysis was performed based on ethnicity (Europeans and Asians). Sensitivity analyses were performed based on HWE (studies without HWE were excluded) and source of controls (hospital-based studies were excluded). Furthermore, Galbraith plot was used to further detect the potential sources of heterogeneity. | Statistical analysis |
| **RESULTS** | | |  |
| Study selection | 17 | The detailed process of study identification was shown in Fig 1. By screening title or abstract and further evaluating full-text, we identified 19 eligible articles from PubMed, Embase, Web of Science, Cochrane database, CBMdisc, CNKI and Google Scholar [4-22]. The baseline characteristics of 19 studies were shown in Table 1 and 2. In total, 4,767 T2DM cases and 10,370 controls were identified to assess the association of the variant with T2DM risk. | Characteristics of the studies |
| Study characteristics | 18 | Table 1 shows the studies included in the meta-analysis and their main characteristics. | Characteristics of the studies |
| Risk of bias within studies | 19 | The funnel plot did not show obvious asymmetry in any genetic model (Fig. 4). In addition, the results of Egger’s regression test did not provide any statistical evidence for publication bias (p = 0.475 for allelic model, p = 0.647 for additive model, p = 0.581 for recessive model, and p = 0.452 for dominant model). Therefore, there was no risk of publication bias in the present meta-analysis. | Publication bias evaluation |
| Results of individual studies | 20 | The main results of individual studies were shown in Fig 2 (Forest plots). | Quantitative synthesis |
| Synthesis of results | 21 | The overall results showed evidence of significant association between the APOA5 -1131T/C polymorphism and T2DM risk, suggesting that the APOA5 -1131T/C polymorphism was a risk factor for T2DM (for C allele vs. T allele: OR = 1.28, 95% CI = 1.17-1.40, p < 0.01; for C/C vs. T/T: OR = 1.57, 95% CI = 1.35-1.83, p < 0.01; for C/C vs. T/C+T/T: OR = 1.36, 95% CI = 1.18-1.57, p < 0.01; for C/C+T/C vs. T/T: OR = 1.32, 95% CI = 1.16-1.51, p < 0.01). The main results of meta-analysis were shown in Table 2 and Fig 2. | Quantitative synthesis |
| Risk of bias across studies | 22 | Begg’s funnel plot and Egger’s regression test were performed to assess potential publication bias. The funnel plot did not show obvious asymmetry in any genetic model (Fig. 4). In addition, the results of Egger’s regression test did not provide any statistical evidence for publication bias (p = 0.475 for allelic model, p = 0.647 for additive model, p = 0.581 for recessive model, and p = 0.452 for dominant model). Therefore, there was no risk of publication bias in the present meta-analysis. | Publication bias evaluation |
| Additional analysis | 23 | **Subgroup analyses:** In the subgroup analysis based on ethnicity, significant association was also found between the APOA5 -1131T/C polymorphism and T2DM risk in Asians (for C allele vs. T allele: OR = 1.31, 95% CI = 1.22-1.40, p < 0.01; for C/C vs. T/T: OR = 1.61, 95% CI = 1.38-1.88, p < 0.01; for C/C vs. T/C+T/T: OR = 1.39, 95% CI = 1.20-1.61, p < 0.01; for C/C+T/C vs. T/T: OR = 1.42, 95% CI = 1.25-1.62, p < 0.01), but not in Europeans (for C allele vs. T allele: OR = 0.91, 95% CI = 0.69-1.20, p = 0.49; for C/C vs. T/T: OR = 0.68, 95% CI = 0.27-1.70, p = 0.41; for C/C vs. T/C+T/T: OR = 0.66, 95% CI = 0.27-1.66, p = 0.38; for C/C+T/C vs. T/T: OR = 0.88, 95% CI = 0.67-1.15, p = 0.34). The main results of subgroup analysis were shown in Table 2.  **Sensitivity analyses:** Sensitivity analyses were respectively performed based on HWE (studies without HWE were excluded) and source of controls (hospital-based studies were excluded). Overall, the pooled ORs and 95% CIs were not materially altered when any part of the study was omitted, indicating that our results were statistically robust. The results of sensitivity analyses were shown in Table 2.  **Heterogeneity analysis:** Signiﬁcant heterogeneity existed in the allelic model (PQ = 0.050, I2 = 38.4%) and dominant model (PQ = 0.004, I2 = 52.3%). In contrast, the additive model (PQ = 0.374, I2 = 6.8%) and recessive model (PQ = 0.692, I2 = 0%) did not present significant heterogeneity. To clarify the sources of heterogeneity, we first conducted the subgroup and sensitivity analyses. We only effectively removed the heterogeneity in the Europeans group. We next created a Galbraith plot to graphically assess the sources of heterogeneity. A total of four studies [8,12,21,22] were identified as the main contributors to heterogeneity (Fig. 3). After excluding the outlier studies, the heterogeneity was effectively removed (data not shown). | Quantitative synthesis, sensitivity analysis and heterogeneity analysis |
| **DISCUSSION** | | |  |
| Summary of evidence | 24 | The overall results showed that there was significant assiciation between the APOA5 -1131T/C polymorphism and the T2DM risk, suggesting the C allele was a independent risk factor for the development of T2DM. The findings showed that the risk of developing T2DM in C allele carriers was 1.32-fold higher than those without. Furthermore, the individuals with C/C genotype had a significantly higher risk for developing T2DM (for OR=1.57 in additive model and OR=1.36 in recessive model) compared to those with T/C genotype and T/T genotype. | Discussion |
| Limitations | 25 | **Limitations:** Firstly, there was significant between-study heterogeneity in the allelic model and dominant model. Heterogeneity is a problem that may affect the precision of overall results. Secondly, in 19 studies included for this meta-analysis, only four of them are European samples, which may develop a partial result. Thirdly, the controls were not uniformly deﬁned. Both healthy individuals and patients without T2DM in hospital were included in the control group. The hospital-based controls may not be representative of the randomized population. Therefore, some inevitable selection bias might be brought into the meta-analyses, which might affect the interpretation of the final results. Moreover, meta-analysis is a type of retrospective study, and recall and selection bias might inevitably exist. | Discussion |
| Conclusions | 26 | **Conclusion：**In conclusion, the present meta-analysis suggests that the APOA5 -1131T/C polymorphism is associated with an increased risk of T2DM, especially in Asians. However, the result should be interpreted with caution because of its limitations. Larger and well-designed studies based on different ethnic samples are needed to confirm our results in the future. | Discussion |
| **FUNDING** | | |  |
| Funding | 27 | N/A. | Online submission system |

*From:*  Moher D, Liberati A, Tetzlaff J, Altman DG, The PRISMA Group (2009). Preferred Reporting Items for Systematic Reviews and Meta-Analyses: The PRISMA Statement. PLoS Med 6(6): e1000097. doi:10.1371/journal.pmed1000097

For more information, visit: **www.prisma-statement.org**.

Page 2 of 2
